# A landscape persistence-based methodological framework for assessing ecological stability

**DOI:** 10.1016/j.ese.2023.100300

**Published:** 2023-07-11

**Authors:** Da Lü, Yihe Lü, Guangyao Gao, Siqi Sun, Yi Wang, Bojie Fu

**Affiliations:** a State Key Laboratory of Urban and Regional Ecology, Research Center for Eco-Environmental Sciences, Chinese Academy of Sciences, PO Box 2871, Beijing, 100085, China; b University of Chinese Academy of Sciences, Beijing, 100049, China

**Keywords:** Ecological stability assessment framework, Landscape changes, Nature conservation and ecological restoration, Nature-based solutions, Qingzang Plateau

## Abstract

Ecological stability is a critical factor in global sustainable development, yet its significance has been overlooked. Here we introduce a landscape-oriented framework to evaluate ecological stability in the Qingzang Plateau (QP). Our findings reveal a medium-high stability level in the QP, with minimal changes over recent years. The driving factors vary across landscape types, with climate and anthropogenic factors emerging as crucial determinants. While anthropogenic factors are strong but unstable due to policy changes and economic development, climatic factors exert a consistent influence. Based on our results, we propose site-specific ecological conservation and restoration measures. The ecological stability assessment framework provides a practical tool to understand the link between environmental conditions and ecosystems.

## Highlights

• Ecological stability can be measured by landscape change probabilities.
• A probability-based Ecological Stability Evaluation Framework (ESEF) is proposed.
• The contribution of factors to ecological stability varies among landscape types.
• The ESEF can be useful in enhancing regional ecological conservation and restoration.

## Graphical abstract

**Figure:**
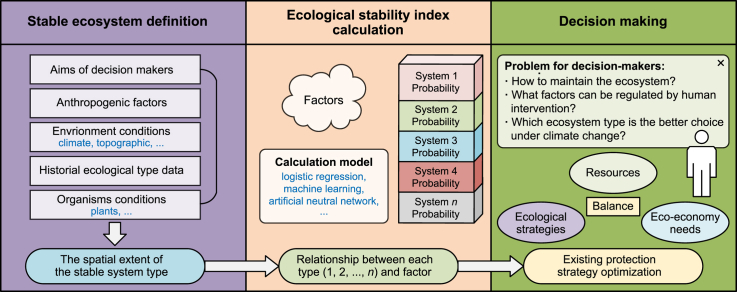

## 1 Introduction

Ecosystem degradation has emerged as a critical global environmental concern due to the continuous progress of industrialization and urbanization [1,2]. In recent years, ecological restoration measures have been widely implemented worldwide to combat land degradation and achieve sustainable development goals [[3], [4], [5], [6], [7]]. The global improvement in ecological quality has demonstrated the benefits of ecological restoration projects and strategies [8]. The United Nations Decade on Ecosystem Restoration aspires to maintain and improve the integrity of diverse ecosystems, offering a practical approach to achieve the sustainable development goal of land degradation neutrality. However, global climate change has resulted in gradual shifts in geographical conditions, imposing growing pressure on ecosystems worldwide [9]. Limited resources further exacerbate the challenge of sustaining restored ecosystems in environmentally hostile areas without human intervention [10,11]. Concerning these impacts, the sustainability of ecosystems is a significant issue for living nature.

Understanding the sustainability of ecosystems relies significantly on comprehending ecological stability [[12], [13], [14], [15]], which signifies the alterations in ecosystem components over time [12,13,16,17]. The study of ecological stability is built upon two major concepts: one emphasizes systems close to equilibrium, and the other focuses on non-equilibrium behavior and the attractiveness of different domains [13,14]. Therefore, there is still no consensus among scientists on the definition of “ecological stability” [13]. Nevertheless, stability indices continue to be widely used in micro- and macro-ecological studies, especially in community stability and landscape stability studies [[18], [19], [20], [21]]. In an ecological evaluation, an increase in the stability index usually indicates the success of an ecological restoration strategy.

Determining and quantifying ecological stability is more feasible and precise in small-scale ecosystems due to the availability of sufficient data. Conversely, large-scale studies face significant challenges in obtaining data, especially in some large and remote regions. Several improvised models have been developed to overcome this problem of data scarcity [2,18,[22], [23], [24]]. However, the factors considered by ecologists in ecological stability assessment models are often influenced by underlying scientific assumptions and data availability [[24], [25], [26]]. Natural resources can be reallocated to meet the ecological and economic development needs of a region through human interventions. However, the flow of resources might also lead to reduced resource utilization in the supply areas. Only those ecosystems that can persist over time, irrespective of whether they are grasslands, forests, or wetlands, can ensure the continued functioning of the entire ecosystem [18]. Therefore, they can be considered the “backbone” of socio-ecological sustainability. A stable ecosystem equipped with self-regulating mechanisms that harmonizes with the environment lays the foundation for achieving sustainable development goals. Thus, there is an urgent need for studies on ecological stability to elucidate the stability characteristics of ecosystems.

The steady state characterized by ecological stability is very useful for early warnings of ecological and environmental concerns [27,28]. History-based extrapolation can provide essential insights into the stability and sustainability of ecosystems. Understanding the environmental conditions required to maintain current ecosystems and the effects of various factors on different ecosystems are extremely important for improving and maintaining ecosystems. Examining the stability of different ecosystems and their relationships with environmental conditions can enhance the realistic value of ecological protection and restoration measures. In this study, we adopt a landscape-oriented perspective to define ecological stability, which captures the steady state among ecosystems. By considering the relationships between environmental conditions and ecosystem types from the landscape context, we propose a framework to address the above problems and identify appropriate metrics for each ecosystem type.

## 2 Methods

### 2.1 Ecological stability from a landscape perspective

A system is considered stable if it can self-regulate to maintain its original state despite environmental stresses [28]. In this study, the concept of "ecological stability" refers to the probability of maintaining terrestrial landscape, which serves as an external feature of ecosystems. Based on the topology and principles of ecological stability [16], a probability-based quantification approach can be developed to assess ecological stability across spatial scales. The ecosystems are deemed more stable if the probability of occurrence of their major ecosystem features is higher.

### 2.2 Ecological stability assessment framework

**Fig. 1:**
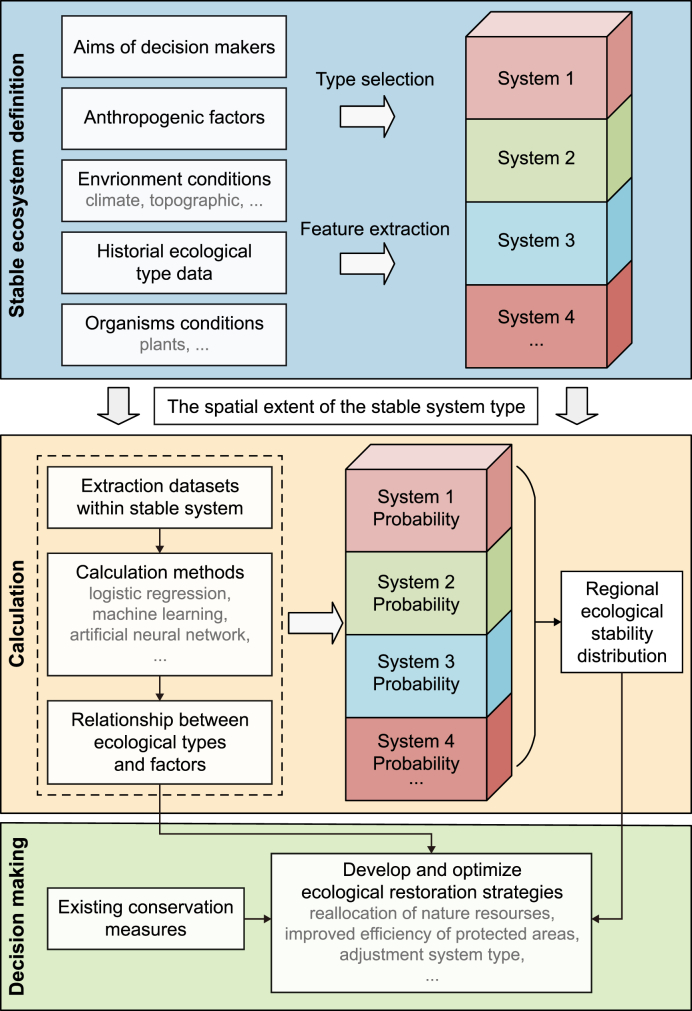
Ecological stability assessment framework.

Ecological stability is a feature of ecosystems and can be used to measure the self-sustaining ability of the ecosystems. From a historical perspective, we proposed a framework based on landscape changes to characterize ecological stability. The framework consists of two major components: the definition of stable ecosystems and the estimates of ecosystem maintenance probabilities (Fig. 1).

The regional landscape system consists of several ecosystems. To elucidate the status of each part, ecosystems were initially categorized into different types based on their structural and functional features. Then, the relationship between the ecosystems and environmental factors can be established using a calculation and analysis module, and the probability of each terrestrial ecosystem can be calculated to define the quantitative relationship (Fig. 1). Further, by integrating the probabilities of each ecosystem, as represented by systems 1–4 in Fig. 1, the ecological stability in the entire region can be quantified. The differences in stability among ecosystems provide valuable insights for policymakers to formulate informed ecological strategies and aid in the exploration of mechanisms underlying ecosystem adaptation to environmental changes.

### 2.3 Application of the ecological stability assessment framework to the Qingzang Plateau

**Fig. 2:**
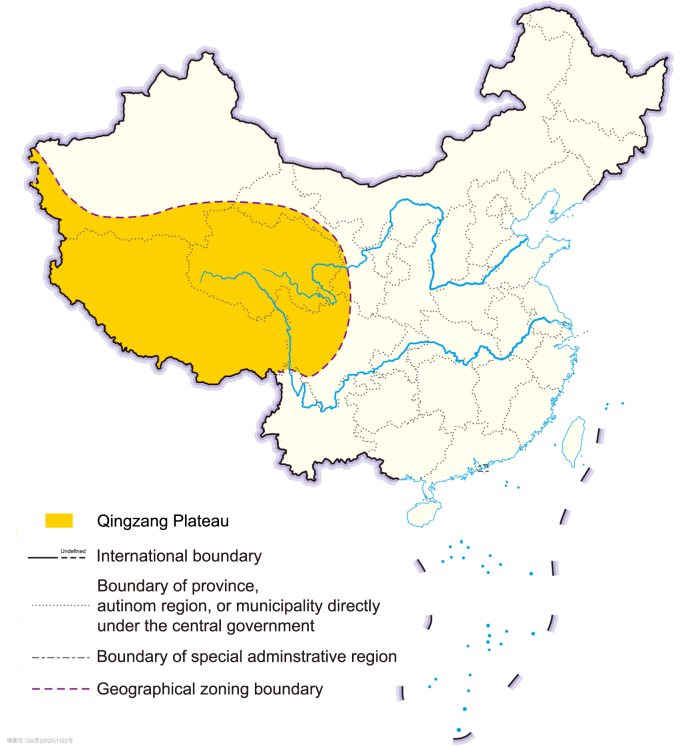
Location of the Qingzang Plateau.

The Qingzang Plateau (QP) holds significant ecological importance in Asia and globally, serving as a vital water tower and critical habitat for wildlife [29,30]. Situated in southwestern China (Fig. 2), it spans an expansive area of 2.6 × 10^6^ km^2^, with an average altitude surpassing 4,000 m [31]. As the largest alpine ecosystem, the meadows in the QP provide valuable ecosystem services [32]. The annual average precipitation and temperature are 413.6 mm and 1.61 °C, respectively. Although the intensity of human activities is increasing, mainly along the mid-eastern regions on the QP, most parts of the region have not witnessed significant human intervention [35]. Since 1963, several ecological conservation measures were undertaken, leading to a gradual regional stabilization of ecosystems on the QP. To date, 171 protected areas have been established, covering 35.5% of the QP's total area [31]. This study focuses on the QP as a crucial ecological site to demonstrate the proposed framework's efficiency.

#### 2.3.1 Definition of stable ecosystems on the QP

The first and most critical step was identifying the stable ecosystems on the QP. For this, the spatial extent of a steady state of each ecosystem type had to be identified. Due to the lack of vegetation-type data, the Land Use and Land Cover (LULC) Level Two Classification datasets were used for differentiating between the zones of change and the zones of persistence [33]. If no change was observed in a certain land cover from 2000 to 2015, the ecosystems of such a land cover were considered to be stable. All the land-cover types closely linked to ecological quality, such as forestlands, grasslands, and wetlands, were selected as surrogates for ecosystem stability analysis. Contrarily, desert and urban land-cover types were not considered because of their low vegetation activity. The zones used for ecological stability assessment were then optimized again by combining the multi-year change characteristics of NDVI and GPP to exclude the errors due to land-cover classification. Finally, the landscape types were identified using cluster analysis based on the land-cover types and vegetation features (Table S1).

#### 2.3.2 Calculation of the ecological stability of the QP

In this study, the environmental factors were selected based on the relevant ecological studies [9,13,34,35]. These factors encompass four aspects: anthropogenic factors (*X*_1_–*X*_2_), climate factors (*X*_3_–*X*_4_), resource availability (*X*_5_–*X*_6_), and topographic (*X*_7_*–X*_9_) and vegetation (*X*_10_–*X*_11_) conditions (Table 1).

**Table 1:** Parameters and data sources used in this study.

Code	Data name	Resolution	Data source
*X*_1_	Gross domestic product (GDP)	1 km	[63]
*X*_2_	Population density	1 km	[64]
*X*_3_	Precipitation	1 km	[65]
*X*_4_	Temperature	1 km	
*X*_5_	Distance to waterways	1 km	From OpenStreetMap (Accessed 8 May 2021) [66]
*X*_6_	Distance to roads	1 km	
*X*_7_	Aspect	90 m	Calculated based on DEM
*X*_8_	Elevation	90 m	SRTM DEM dataset [67,68]
*X*_9_	Slope	90 m	Calculated based on DEM
*X*_10_	Gross primary productivity (GPP)	0.05°	[69]
*X*_11_	Normalized difference vegetation index (NDVI)	1 km	[70]
-	Land use and land cover	1 km	[71]
-	Enhanced vegetation index (EVI)	1 km	[72]

Note: The data are for the years 2000, 2005, 2010, and 2015, and all data are resampled to 1 km for calculation.

For all landscape types, the landscapes were either in a steady state (1) or an unsteady state (0). If a particular landscape type remained consistent throughout the study period, it was considered a steady system. The sample grid point datasets, including *X*_1_–*X*_11_, were extracted based on the spatial extent of the steady landscape system. All factors were standardized as follows:

$$Z_{i}=\frac{X_{i}-X_{i,\min}}{X_{i,\max}-X_{i,\min}}$$

where *Z*_*i*_ is the normalized factor of *X*_*i*_. *X*_*i*,min_ and *X*_*i*,max_ denote the minimum and maximum values of factor *X*_*i*_, respectively.

In this study, logistic regression, a widely employed analytical method [[36], [37], [38]], was employed to build the relationship between the landscape and environmental factors and compute the probabilities of each landscape type. The probabilities of each landscape are defined as follows:

(1) $$\text{Log}\left(\frac{P_{i}}{1-P_{i}}\right)=\beta_{0}+\beta_{1}Z_{1}+\beta_{2}Z_{2}+\cdots +\beta_{n}Z_{n}$$

(2) $${\text{ESI}}_{i}=P_{i}=\frac{e^{\beta_{0}+\beta_{1}Z_{1}+\beta_{2}Z_{2}+\cdots +\beta_{n}Z_{n}}}{1+e^{\beta_{0}+\beta_{1}Z_{1}+\beta_{2}Z_{2}+\cdots +\beta_{n}Z_{n}}}$$

where *P*_*i*_ is the probability of the *i*th landscape, denoted as the ecological stability index (ESI) of the *i*th landscape (ESI_*i*_), which indicates the degree of ecological stability of the *i*th landscape. The range of ESI_*i*_ is 0–1, with a larger value of ESI_*i*_ indicating greater ecological stability of the landscape. *Z*_*i*_ indicates the normalized environmental factors associated with ecological stability. The coefficients (*β*) are estimated using logistic regression, considering the landscape pattern as a dependent variable.

Finally, the spatial distribution of ecological stability at the landscape scale on the QP was mapped by computing the ESI_*i*_ using the Maximum Value Composite (MVC) method (equation (3)) [39]:

(3) $$\text{ESI}=\max\left\{{\text{ESI}}_{1},{\text{ESI}}_{2},\ldots ,{\text{ESI}}_{n}\right\}$$

where *n* represent the total number of categories, and ESI indicates the ecological stability of the region. The spatial patterns were calculated using a multi-year ESI average, and the ESI was divided into five classes: very high ecological stability (0.8–1), moderately high ecological stability (0.6–0.8), medium ecological stability (0.4–0.6), low ecological stability (0.2–0.4), and very low ecological stability (0–0.2). Additionally, the trend was determined using linear regression in MATLAB.

## 3 Results

### 3.1 Spatial patterns and temporal changes of landscape ecological stability in the QP

**Fig. 3:**
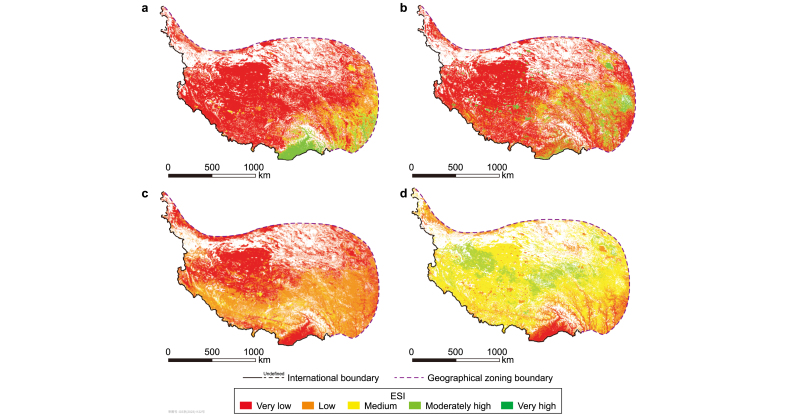
The ecological stability (ESI) of forests (**a**), wetlands (**b**), high-coverage grasslands (**c**), and sparse grasslands (**d**).

Each landscape of the QP showed various patterns of ecological stability. The forests with high ecological stability levels accounted for 6.87% of the QP, with 3.82% classified as very high stability and 3.06% as moderately high stability. These forests were primarily concentrated in the southeast (Fig. 3a). The wetlands with high ecological stability levels were also spatially concentrated. The areas with very high ecological stability and moderately high ecological stability accounted for 3.97% and 4.42% of the QP, respectively (Fig. 3b). The ESI of high-coverage grasslands and sparse grasslands, as the primary landscape components of the QP, had different spatial patterns (Fig. 3c,d). The high-coverage grasslands were concentrated only in the south of QP, and the areas with high ecological stability levels accounted for 0.2%. The areas with high ecological stability levels in sparse grasslands accounted for 12.83%; however, the areas with moderately high ecological stability accounted for 12.74%. The high-coverage grasslands (40.89%) and sparse grasslands (20.97%) had larger areas with low ecological stability levels. Notably, sparse grasslands exhibited the largest area (60.98%) at medium ecological stability among all landscapes, indicating their wider distribution due to less stringent environmental requirements.

**Fig. 4:**
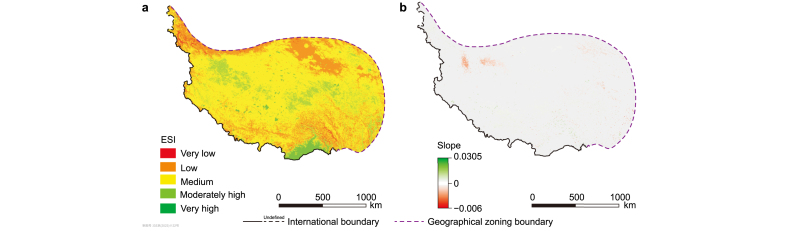
The spatial pattern (**a**) and significant change trend (**b**) of ecological stability (ESI) at confidence levels of 0.05 from 2000 to 2015 on the Qingzang Plateau.

The areas with very high and moderately high ecological stability accounted for 12.91% of the QP, whereas 65.56% of the QP was a region of medium stability (Fig. 4a). The ecological stability did not show a significant change in trend in most of the QP during 2000–2015. The areas with a significant increase and decrease in ecological stability accounted for 1.27% and 0.70% of the QP, respectively, during this time period (Fig. 4b). The regions with significantly reduced stability were mainly located in the sparse grasslands and permanent glacial areas in inland alpine regions, probably due to rising temperature [40]. Fortunately, the magnitude of variation in ecological stability was limited, with a maximum reduction in a slope of 0.006, indicating no obvious tendency of change in the ecological type during the study period. However, this trend should not be ignored, as the increasing global warming may lead to escalating ecological security problems for alpine ecosystems in the QP [41].

### 3.2 Relationship between the driving factors and the ecological stability at the landscape scale

**Fig. 5:**
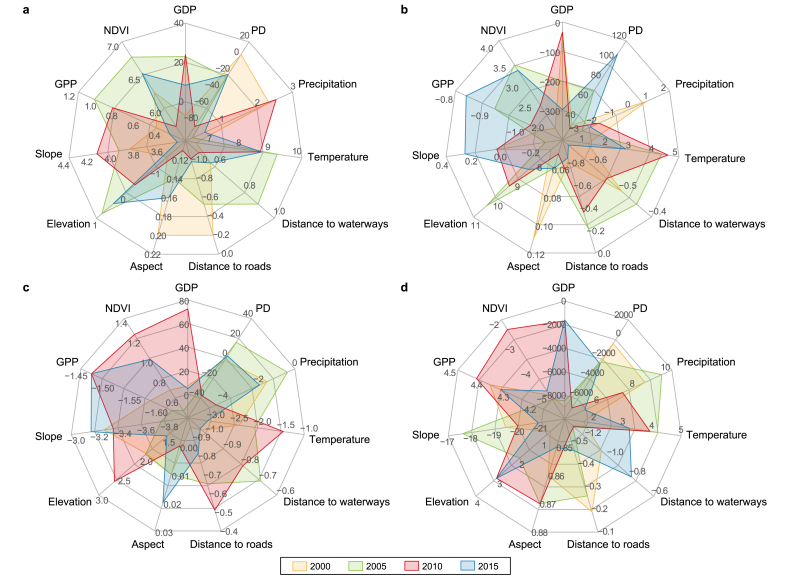
The contribution of factors in forests (**a**), wetlands (**b**), high-coverage grasslands (**c**), and sparse grasslands (**d**) from 2000 to 2015.

There is a variation in the contribution of environmental driving factors to the ecological stability of each landscape type (Fig. 5). Among these driving factors, climate and anthropogenic factors were found to be the most significant (Table S2). The anthropogenic factors demonstrated large and temporal variations in the contributions to each landscape type. For example, in the year 2000, gross domestic product (GDP) has a negative contribution, while population density had a positive contribution to the forest landscapes. But in other years, these two factors showed opposite contributions. This implies that ecological restoration projects have been largely beneficial in improving human behavior toward ecological conservation [35]. The population density trend also highlighted the same fact that conservation policies (Table S3) promoted human settlements to move away from forests [42,43]. On the other hand, both the grassland types failed to build a balanced relationship with anthropogenic factors during the study period. However, sparse grasslands, in general, are less sensitive to human activities due to harsh conditions that limit widespread human habitation [44].

During the study period, the impact of natural factors remained relatively stable. Temperature and precipitation were important primary external conditions for all landscape types; however, their effects on ecological stability varied. In the forest landscape types, temperature had a greater positive influence compared to precipitation from 2000 to 2015, with a slight increase trend observed for the contribution of temperature. The wetlands of the QP were positively influenced mainly by precipitation, but with the increase in glacier melt water resulting from rising temperatures, the contribution of temperature to the ecological stability of wetlands also gradually increased [45]. Grasslands differed from the above two landscape types, as temperature and precipitation did not always have a positive effect on the stability of grasslands. Excessive warming and precipitation can disrupt the original environmental conditions, leading to an imbalance in the ecosystem, decreased grassland stability, and the initiation of ecological succession [46]. Vegetation and topographic conditions were key drivers of landscape changes; for example, elevation was a factor with a higher contribution to the ecological stability of high-coverage grasslands, while gross primary productivity (GPP) had a stable positive contribution to the ecological stability of forest landscapes. Overall, this study highlighted the factors influencing landscape adaptation to environmental conditions and their role in maintaining stability. Moreover, it aids in identifying critical factors for ecological maintenance in each landscape type.

## 4 Discussion

### 4.1 Application of the ecological stability assessment framework

Due to the limited data on the QP, a two-step validation was used to ensure the reliability of the method. First, the model was validated by comparing the fit of the actual land-surface features with the ESI (Fig. S1). Four surface features showed a strong alignment with the different ESI levels. The enhanced vegetation index (EVI) was used for quantifying vegetation greenness, as it has been established as an indicator of terrestrial ecosystems and land surface situations [47]. The results of Pearson correlation analysis between EVI and ESI indicated a significant positive correlation (Table S4). This suggested that the developed ESI can effectively characterize the stability level of the different landscape types of the QP. Notably, the results in Table S2 highlight the differences in the roles of various environmental factors on ecological stability. Unlike natural factors, anthropogenic factors exhibited greater volatility yet still exerted a critical impact on the ecological stability of the QP. In addition, the spatial distribution of forests, grasslands, and wetlands of the QP was consistent with the spatial distribution of ESI.

Studies on ecological stability have focused mainly on community and landscape stability [13,21,24]. Regional-scale assessment studies often belong to the category of landscape stability. However, assessing landscape stability at a regional scale using models is challenging when data availability is limited [2,23]. Consequently, researchers commonly resort to basic methods, such as the coefficient of variation [48] and the coefficient of ecological importance [18], for landscape stability assessment. However, these methods lack a common clear standard, making it difficult to verify the accuracy of ecological stability indicators. Therefore, these methods have a lower application-oriented value [13,14,18,22]. The major focus of this study was to develop an easy-to-use methodological tool for assessing ecological stability regardless of data insufficiency in large and remote regions. Considering the limitations of data on the QP, a longer time series and additional variables were not considered in this study. Nevertheless, the framework based on historical data from the perspective of a landscape has the potential for ecological stability assessment. Firstly, it enables the identification of distinct macro-scale ecological landscape types, providing a uniform standard for comparing different studies on ecological stability. Secondly, the equilibrium of an ecosystem was determined based on environmental conditions rather than time, allowing datasets from different times pointed to be combined for analyzing the fundamental mechanisms driving the maintenance of the ecosystem at the landscape scale. Lastly, a thorough understanding of the above mechanisms can be useful to determine the types of ecosystems with high stability or landscapes that require conservation or restoration based on environmental conditions.

### 4.2 Undertaking conservation and restoration based on ecological stability

**Fig. 6:**
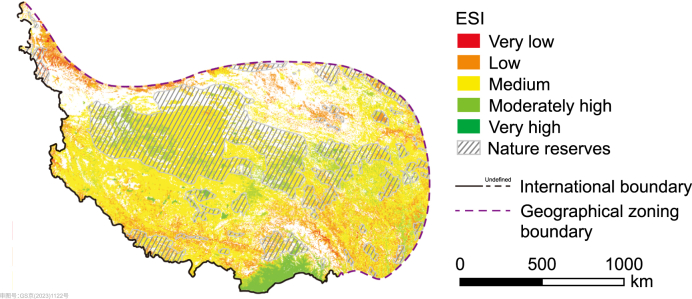
Spatial distribution of extant nature reserves and ESI on the Qingzang Plateau.

The QP is a complex system with a long history, with human presence dating back approximately 160,000 years ago [49]. The central region of the QP experienced an uplift around 21–26 million years ago, reaching an elevation of 3500–4500 meters [50]. Overall, the QP exhibits remarkable stability with limited human activity [35]. This indicates that the long-term effect of human intervention on ecological restoration appears to be weak. In this study, the average ecological stability was observed to be higher in areas with nature reserves (0.54) than in areas without reserves (0.47), with 17.95% of areas with reserves demonstrating high ecological stability. The areas without nature reserves accounted for about 10.79% (Fig. 6). These results demonstrated the positive effect of nature conservation on ecological stability during the study period. However, ecological conservation with human interventions needs financial support. Due to the extensive size of the QP, conserving the entire area would necessitate an impractical level of investment [51]. In recent years, the increasing focus of ecological conservation is on the core functions and services to enhance cost-effectiveness [[51], [52], [53]]. Contrary to previous studies that focused on ecological risks or functions, this study proposed the use of ecological stability methods for decision-making on practical conservation or restoration projects. The region with high ecological stability is considered to be relatively healthy without any human interference. For example, the forests of the southeastern QP are extremely stable, and community forests [54] could be utilized as an alternative to the costly nature reserve strategy, promoting the harmonization of the utilization and protection of relevant ecosystems and natural resources. Special emphasis should be placed on implementing ecological conservation measures in the regions of the QP characterized by moderate ecological stability, encompassing approximately 64.07% of the area. These regions are particularly vulnerable to the impacts of human activities and climate change. Therefore, it is crucial to focus on ecological protection or restoration measures to enhance the stability of these areas and establish stable and balanced ecosystems. On the one hand, this can save conservation costs and avoid ineffective or excessive protection measures and interventions [10]. On the other hand, in situations where ecological degradation is challenging to halt through human interventions, proactive planning of future ecological states becomes crucial. This can be achieved by applying passive (or natural) ecological restoration measures. For example, global warming exceeding 1.5 °C can lead to a collapse of the ice sheets and widespread permafrost thaw [55], which can drastically alter the soil and water conditions on the QP. Elucidating the living environment of each ecosystem type can help to undertake natural succession as a control measure to plan for unpredictable future scenarios.

Achieving global ecosystem sustainability does not necessarily require massive financial investments to align environmental conditions with desired ecosystem types. Instead, it can be accomplished by establishing ecosystem types that are compatible with the existing environment. Despite the implementation of numerous ecological measures (Table S3), 5.23% of the nature reserves of the QP are still in the low and very low ecological stability levels (Fig. 6). This might be due to the poor matching between ecological measures and local environment, such as the enhanced degradation of grasslands due to the implementation of the Nomad Settlement Policy [42] and the increased potential risk of water resources due to afforestation [10]. In addition, achieving a harmonious balance between ecological conservation, restoration, and livelihood development on the QP is very important for sustainable development [[56], [57], [58], [59]]. With socioeconomic development, the increased pressure of human interference for supporting livelihoods has led to ecological problems, such as grassland degradation due to overgrazing [60] and decreased regional connectivity due to road construction [58]. However, implementing strict protection measures can restrict such development, potentially giving rise to livelihood problems [56,61]. Therefore, ecosystem integrity needs to be considered in ecological conservation and restoration strategies [62]. The maintenance of optimal surface landscapes can ensure a balance between conservation and development in the overall region. The ecological stability assessment framework developed in this study provides a methodology for selecting the optimal landscape type based on the principles of nature-based solutions. The ecological conservation and restoration of the QP also should be tailored to suit the specific environmental conditions of each ecological landscape type, thus preventing ecosystem imbalances and regional-scale degradation.

## 5 Conclusion

Stable ecosystems are indispensable components of the socio-ecological systems for achieving global sustainable development. However, the lack of consistent concepts and scientific standards has hindered research and practical applications concerning ecological stability. To address this gap, this study introduces a landscape-based ecological stability assessment framework, which is applied in the Qingzang Plateau (QP) in China. The results indicated the effectiveness and applicability of this framework, particularly in large and remote areas. This research tries to explore the comprehensive relationships between environmental conditions and ecosystems. The existing forests, grasslands, and wetlands in the QP show a strong consistency with areas of high ecological stability. The entire QP belongs to a medium to high ecological stability region, with minimal changes observed in the ecological stability over the past few decades. The contribution of driving factors to ecological stability varies according to the regional landscape types. While anthropogenic factors exert a strong but highly volatile influence due to changes in policy and economic development. In contrast, climatic factors provide a continuous and stable effect. Spatially explicit mapping and variational trends in ecological stability at a regional scale provide valuable information for decision-making on the ecological conservation and restoration of landscapes. Nature-based solutions of conservation are recommended for landscapes with high ecological stability while prioritizing natural restoration for landscapes experiencing declining ecological stability.

## CRediT authorship contribution statement

**Da Lü**: Conceptualization, Methodology, Software, Formal Analysis, Writing - Original Draft. **Yihe Lü**: Conceptualization, Writing - Review & Editing. **Guangyao Gao**: Writing - Review & Editing. **Siqi Sun**: Software, Formal Analysis. **Yi Wang**: Data Curation. **Bojie Fu**: Writing - Review & Editing.

## Declaration of competing interest

The authors declare that they have no known competing financial interests or personal relationships that could have appeared to influence the work reported in this paper.
