# Supplementary material for: A landscape persistence-based methodological framework for assessing ecological stability
Source: Environ Sci Ecotechnol. 2023 Jul 11;17:100300. doi: 10.1016/j.ese.2023.100300 (PMC10407666; doi:10.1016/j.ese.2023.100300)
Supplement: Multimedia component 1 [file mmc1.docx]

**Supplementary material**

**Table.S1 The key features of landscape types on the Qingzang Plateau.**

| Landscape type | Description of main features |
| --- | --- |
| Forest | complex ecological system in which trees are the dominant life-form |
| High-coverage grassland | Covering more than 50% of natural grassland, improved grassland and mowed grassland |
| Sparse grassland | Natural and improved grasslands with a coverage of 5 to 50% |
| Wetland | Marsh, swamp, or other area of land where the soil near the surface is saturated or covered with water |

**Table.S2 The logistic regression coefficient of factors for each landscape type in 2000, 2005, 2010, and 2015.**

| 2000 | Forest | High-coverage grassland | Sparse grassland | Wetland |
| --- | --- | --- | --- | --- |
| Constant | -9.9 | -6.91 | 0.49 | -5.08 |
| GDP | -7.05 | -40.17 | 7.07 | -8988.1 |
| Population density | 4.79 | 35.07 | -2.11 | -385.44 |
| Precipitation | 2.34 | 0.74 | -1.56 | 8.49 |
| Temperature | 6.37 | 1.09 | -2.4 | 1.64 |
| Distance to waterways | 0.57 | -0.5 | -1.02 | -1.07 |
| Distance to roads | -0.2 | -0.8 | -0.95 | -0.19 |
| Aspect | 0.2 | 0.11 | 0.01 | 0.86 |
| Elevation | -1.39 | 7.51 | 1.93 | 0.65 |
| Slope | 3.88 | 0.07 | -3.28 | -19.47 |
| GPP | 0.47 | -1.04 | -1.58 | 4.38 |
| NDVI | 6.24 | 1.79 | 0.68 | -5.04 |
|  |  |  |  |  |
| 2005 | Forest | High-coverage grassland | Sparse grassland | Wetland |
| Constant | -12.25 | -9.99 | 0.15 | -6.79 |
| GDP | 23.00 | -201.57 | - | -8445.5 |
| Population density | -22.88 | 69.00 | 16.16 | -2232.97 |
| Precipitation | 0.20 | -2.89 | -0.41 | 9.43 |
| Temperature | 9.15 | 4.25 | -2.06 | 4.18 |
| Distance to waterways | 0.89 | -0.45 | -0.69 | -1.33 |
| Distance to roads | -0.53 | -0.21 | -0.65 | -0.26 |
| Aspect | 0.14 | 0.06 | 0.01 | 0.87 |
| Elevation | 0.82 | 10.42 | 1.95 | 2.03 |
| Slope | 3.97 | -0.28 | -3.77 | -17.62 |
| GPP | 1.05 | -0.91 | -1.62 | 4.21 |
| NDVI | 6.77 | 3.39 | 0.47 | -5.21 |
|  |  |  |  |  |
| 2010 | Forest | High-coverage grassland | Sparse grassland | Wetland |
| Constant | -10.94 | -9.34 | -0.44 | -7.14 |
| GPD | 23.99 | -34.36 | 72.34 | -1671.36 |
| Population density | -82.37 | 31.92 | -37.64 | -6878.58 |
| Precipitation | 2.54 | -1.95 | -4.41 | 7.24 |
| Temperature | 8.57 | 4.53 | -1.45 | 3.92 |
| Distance to waterways | 0.49 | -0.55 | -0.79 | -1.32 |
| Distance to roads | -1.01 | -0.36 | -0.51 | -0.53 |
| Aspect | 0.11 | 0.05 | - | 0.87 |
| Elevation | -0.3 | 9.39 | 2.65 | 3.07 |
| Slope | 4.16 | 0.05 | -3.36 | -19.98 |
| GPP | 0.88 | -1.01 | -1.47 | 4.43 |
| NDVI | 5.72 | 2.37 | 1.24 | -2.39 |
|  |  |  |  |  |
| 2015 | Forest | High-coverage grassland | Sparse grassland | Wetland |
| Constant | -11.84 | -8.26 | 0.94 | -6.55 |
| GDP | 8.4 | -297.59 | 4.66 | -1604.53 |
| Population density | -19.45 | 106.54 | 2.39 | -2461.59 |
| Precipitation | 0.54 | -2.43 | -1.98 | 5.12 |
| Temperature | 8.62 | 2.72 | -3.07 | 3.22 |
| Distance to waterways | 0.57 | -0.68 | -1.03 | -0.8 |
| Distance to roads | -0.98 | -0.84 | -0.81 | -0.46 |
| Aspect | 0.16 | 0.06 | 0.02 | 0.85 |
| Elevation | 0.43 | 8.53 | 1.47 | 3.08 |
| Slope | 3.47 | 0.27 | -3.17 | -20.86 |
| GPP | 0.35 | -0.83 | -1.47 | 4.34 |
| NDVI | 6.52 | 3.25 | 0.99 | -4.87 |

**Note: "-" indicates that the factor has no significant relationship with the landscape type (p<0.05).**

**Table.S3 Major programs for constructing ecological security shelters of the Qingzang Plateau.**

| Year | Major ecological project |
| --- | --- |
| 2005 | General Plan for Ecological Conservation and Construction of Sanjiangyuan Nature Reserve, Qinghai Province |
| 2007 | Eco-Environment Protection and Comprehensive Management Plan of Qinghai Lake Basin |
| 2009 | Protection and Construction of Ecological Security Shelter Plan in Tibet (2008–2030) |
| 2011 | Regional Ecological Conservation and Environmental Protection Plan of Qinghai-Tibetan Plateau (2011–2030) |
| 2021 | Major Project Construction Plan for Ecological Protection and Restoration of Qinghai-Tibet Plateau Ecological Shelter Area (2021-2035) |

**Table.S4 The correlation coefficients between enhanced vegetation index and ecological stability index.**

| Year | correlation coefficients | Significance |
| --- | --- | --- |
| 2000 | .279^**^ | 0.00 |
| 2005 | .097^**^ | 0.01 |
| 2010 | .272^**^ | 0.00 |
| 2015 | .350^**^ | 0.00 |
| Average of 2000-2015 | .315^**^ | 0.00 |
| Standard deviation from 2000-2015 | .116^**^ | 0.00 |


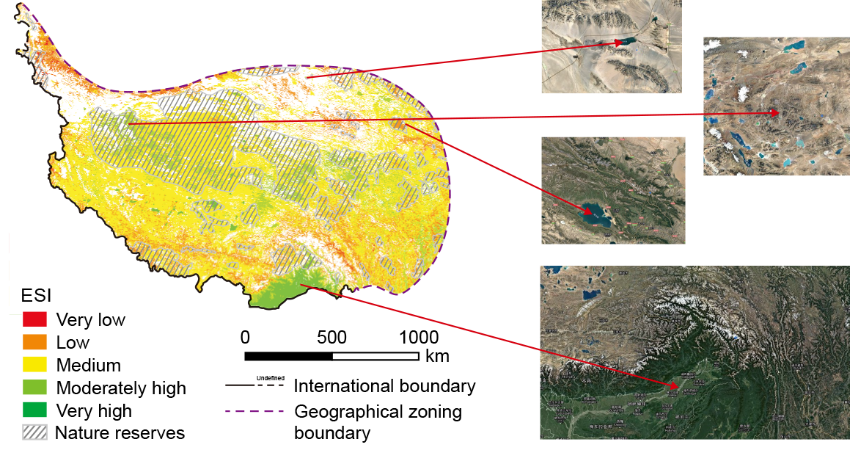


**Figure.S1 Map satellite imagery of verification point in Google Earth.**

Note: very high ecological stability (ESI: 0.8-1); moderately high ecological stability (ESI: 0.6-0.8); medium ecological stability (ESI: 0.4-0.6); low ecological stability (ESI: 0.2-0.4); very low ecological stability (ESI: 0-0.2).

**
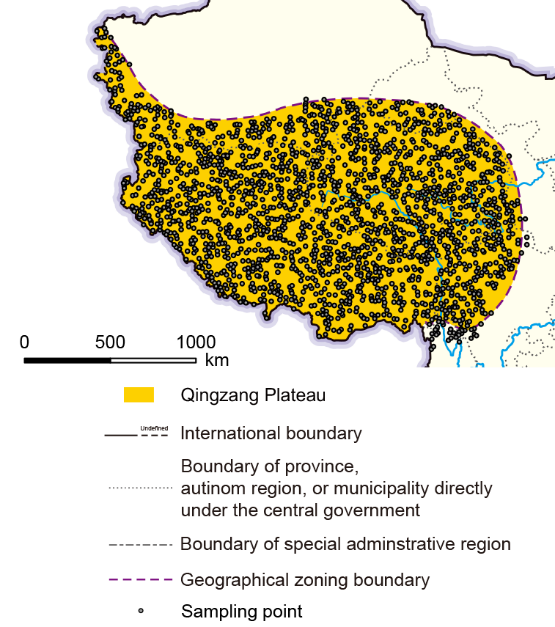
**

**Figure.S2** Spatial location of sampling points for validation.

Note: All sampling points were generated using the ArcGIS Tools-*Creat Random Points*.

The sampling points were used to extract the EVI and ESI for Pearson correlation analysis.
